# Mulberry Leaf Powder Supplemented with a Multi-Enzyme Premix Affects the Intestinal Microbiota–Metabolite–Immunity Axis and Nrf2/Keap1 Signaling in Juvenile *Acipenser schrenckii*: A Dose-Dependent Evaluation

**DOI:** 10.3390/foods15183319

**Published:** 2026-09-19

**Authors:** Wanwan Zhu, Nan Zhang, Xuekai Wang, Yi Xiong, Fuyu Yang, Yongsheng Wang

**Affiliations:** 1 Department of Grassland Science, College of Animal Science, Guizhou University, Guiyang 550000, China; wan850793534@126.com (W.Z.); zn2580042898@126.com (N.Z.); 2 Beijing Tianyuanyugang Farm Co., Ltd. (Beijing Amur Sturgeon Elite Breeding Base), Beijing 101400, China; 3 College of Grassland Science and Technology, China Agricultural University, Beijing 100193, China; xkwang2016@163.com; 4 Frontier Technology Research Institute of China Agricultural University in Shenzhen, Shenzhen 518000, China; xiongleslie@126.com; 5 Shenzhen Branch, Guangdong Laboratory of Lingnan Modern Agriculture, Key Laboratory of Livestock and Poultry Multi-Omics of MARA, Agricultural Genomics Institute at Shenzhen, Chinese Academy of Agricultural Sciences, Shenzhen 518000, China

**Keywords:** enzymatically treated mulberry leaf, *Acipenser schrenckii*, intestinal homeostasis, microbiota–metabolite–immunity axis, Nrf2/Keap1 signaling, aquafeed, dose-dependent response

## Abstract

Mulberry leaf is a promising plant feed resource for aquaculture, yet high inclusion limits its application due to anti-nutritional factors. Exogenous enzyme supplementation can improve the utilization efficiency of bioactive compounds in mulberry leaf-containing diets. This study aimed to evaluate the dose-dependent effects of mulberry leaf powder supplemented with a multi-enzyme premix (MLE) on growth, intestinal health, antioxidant and immune responses, as well as intestinal microbiota and metabolome in juvenile *Acipenser schrenckii*. Four diets containing 0%, 2%, 4% and 6% MLE were formulated for a 10-week feeding trial. Moderate MLE supplementation maintained normal growth without significantly improving growth performance relative to the control, and was associated with reduced serum triglyceride, total cholesterol and glucose, as well as improved intestinal morphology and digestive enzyme activity. Appropriate MLE supplementation was associated with changes in the Nrf2/Keap1 signaling pathway, enhanced antioxidant capacity, and upregulated anti-inflammatory gene *il10*. Multi-omics analysis revealed that MLE induced taxon-specific changes in the intestinal microbiota and was associated with altered metabolic profiles, enriching beneficial taxa including *Priestia*, *Bacillus_P* and *Cetobacterium_A*. These changes suggest a potential modulation of the microbiota–metabolite–immunity axis that may help sustain intestinal homeostasis. Nevertheless, the beneficial effects declined at the 6% inclusion level. Collectively, MLE was associated with dose-dependent protective effects on the intestinal barrier that may involve the microbiota–metabolite–immunity axis and the Nrf2/Keap1 pathway; the optimal supplementary range is preliminarily estimated at 3.5–4.5% for juvenile *Acipenser schrenckii*, although this estimate is based on four dietary levels and requires validation in future dose–response studies.

## 1. Introduction

Sturgeons (order Acipenseriformes) are among the most ancient lineages of extant vertebrates, with origins tracing back approximately 250 million years to the Jurassic period. Often regarded as “living fossils” [1], they hold exceptional value for evolutionary biology. Beyond their scientific importance, sturgeons are highly prized in the food industry: their meat is valued for its palatability and nutritional density, being rich in vitamins, essential amino acids, and minerals that support skin repair, metabolic regulation, and blood pressure modulation. Moreover, sturgeon roe is processed into premium caviar, often termed “black gold” due to its high market value [2,3]. *Acipenser schrenckii* is a rare and economically valuable sturgeon species endemic to China.

Over the past four decades, intensifying market demand for caviar has driven unregulated fishing, illegal trade, and overexploitation. Coupled with anthropogenic pressures such as hydraulic infrastructure, river channel modification, and habitat degradation, these factors have precipitated a continuous global decline in wild sturgeon populations, with most species now classified as endangered [4,5]. In response, large-scale commercial sturgeon aquaculture has expanded rapidly since the late 20th century, with global farmed production now surpassing wild catches [6]. Current estimates indicate annual aquaculture outputs of approximately 120,000 tons of sturgeon meat and 700 tons of caviar. Worldwide, at least 2329 commercial sturgeon farms operate, of which 54% are in China and 24% in Russia [7,8]. China has emerged as the leading producer of farmed sturgeon caviar, exporting 275.83 tons in 2023 [9].

In intensive aquaculture systems, economic viability hinges on feed efficiency, with feed costs constituting 50–60% of total production expenses. The persistently high and volatile prices of high-quality animal protein sources, particularly fishmeal, continue to erode farmer profitability [10]. To reduce unit costs, high-density rearing has become widespread; however, this practice increases disease susceptibility. Antibiotics are frequently incorporated into feeds for disease prevention and growth promotion, which may induce antibiotic residues, microbial resistance, and reduced aquatic product safety [11]. Consequently, there is an urgent need to develop green, efficient, plant-derived feed ingredients as sustainable alternatives to fishmeal and antibiotic growth promoters.

Mulberry leaf is a traditional medicinal and edible plant resource in China and has been approved as a safe feed ingredient [12,13]. It is abundantly available and contains numerous natural bioactive compounds, including plant polysaccharides, flavonoids, and polyphenols, which possess antioxidant and immunomodulatory properties [14,15]. Recent aquatic studies have confirmed that mulberry leaf can improve fish antioxidant capacity, immune status, and intestinal health, displaying great potential as a functional and sustainable fishmeal alternative for aquatic food production [16,17,18].

Mechanistically, the bioactive components in mulberry leaf—particularly polyphenols and flavonoids—are increasingly recognized as modulators of the gut microbiota and its metabolic output. Dietary polyphenols can selectively promote the proliferation of beneficial taxa (e.g., *Lactobacillus*, *Bifidobacterium*) while suppressing pathogenic bacteria, thereby shifting the microbial community toward a health-associated configuration [19]. This microbial remodeling is accompanied by altered metabolite profiles, most notably increased production of short-chain fatty acids (SCFAs) such as butyrate and acetate [20]. SCFAs strengthen the intestinal epithelial barrier, regulate inflammatory cytokine expression, and promote mucosal immunity through G-protein-coupled receptor (GPR41/43) signaling and histone deacetylase (HDAC) inhibition [21], collectively constituting a “microbiota–metabolite–immunity axis.” Notably, the butyrate-sensing machinery and its immunomodulatory effects have been identified in salmonid fish, where butyrate downregulates pro-inflammatory cytokines (IL-1β) and upregulates anti-inflammatory cytokines (IL-10, TGF-β) [22], suggesting that this axis is functionally conserved across teleosts. In parallel, polyphenols and their microbial-derived metabolites can dissociate the Nrf2–Keap1 complex, promoting Nrf2 nuclear translocation and transcriptional activation of downstream antioxidant enzymes (e.g., HO-1, NQO1, SOD, CAT) [23,24]. The resulting enhancement of cellular antioxidant defense mitigates reactive oxygen species accumulation and oxidative damage—a pathway of particular relevance for carnivorous fish under intensive culture, where oxidative stress is a primary driver of immune suppression [25,26]. We therefore hypothesize that mulberry leaf powder supplemented with a multi-enzyme premix (MLE), by increasing the bioavailability of these bioactive fractions, may concurrently modulate the microbiota–metabolite–immunity axis and be associated with changes in the Nrf2/Keap1 pathway to improve sturgeon physiological health.

However, natural mulberry leaf contains tannins and phytic acid, which inhibit digestive enzyme activity and nutrient utilization, limiting its effective application in high-protein sturgeon diets [27]. Exogenous enzyme supplementation can effectively degrade anti-nutritional factors, break plant cell walls, and release bound nutrients and bioactive components, thereby improving feed digestibility and intestinal physiological function of fish [28,29]. Metabolomics has also proven to be a valuable tool for assessing biochemical variation and molecular quality traits in fish, supporting its application in aquaculture nutrition studies [30]. Despite the growing understanding of mulberry leaf application in aquaculture, most studies focus on common freshwater fish, while systematic investigations regarding mulberry leaf powder supplemented with a multi-enzyme premix (MLE) in sturgeon—a high-value, carnivorous fish species with high nutritional requirements and pronounced susceptibility to oxidative stress and intestinal dysfunction under intensive culture [31,32]—remain insufficient. Whether dietary MLE supplementation can modulate the gut microbiota–metabolite profile, be associated with changes in the Nrf2/Keap1 antioxidant pathway, and thereby improve immune status and intestinal health in Amur sturgeon remains unclear.

Therefore, this study systematically investigated the effects of graded dietary MLE supplementation on the growth performance, serum metabolism, antioxidant capacity, immune-related parameters, and intestinal health of farmed *A. schrenckii*. To test the proposed mechanistic hypothesis, we examined the gut microbiota composition by 16S rRNA sequencing, profiled intestinal metabolites by LC-MS metabolomics, measured the mRNA levels of key immune cytokines including *il-1β*, *il-10*, *tgf-β*, and *tnfa*, and assessed the expression of Nrf2/Keap1 pathway-related genes including *nrf2*, *keap1*, and *cat*. The present work aimed to clarify the preliminary MLE inclusion level and its functional mechanism, with particular attention to the microbiota–metabolite–immunity axis and the Nrf2/Keap1 signaling pathway. The results are expected to provide a scientific basis for developing a practical, low-cost, and functional plant-based feed strategy for sturgeon culture and to offer a reference for future studies exploring plant-derived feed additives as potential alternatives to antibiotic growth promoters in intensive aquaculture.

## 2. Materials and Methods

### 2.1. Animal Ethics

All experiments were approved by the Inspection Form for the Guizhou University Experimental Animal Ethics (approval No. EAE-GZU-20240E072).

### 2.2. Diets, Animals and Experimental Design

Four experimental diets (CK, MLE2, MLE4, MLE6) were formulated. The control diet (CK) contained no mulberry leaf powder, while the three experimental diets included 2.0%, 4.0% and 6.0% sun-dried mulberry leaf powder, respectively. Sun-dried mulberry leaves were purchased from Shichao Fruit and Vegetable Planting Farmers Professional Cooperative (Shangqiu, Henan, China) and pulverized into powder using a small mill. A multi-enzyme premix (0.2%; containing xylanase, cellulase, glucanase and other functional enzymes, Guangdong VTR Bio-Tech Co., Ltd., Zhuhai, China) was supplemented only to the three mulberry leaf-containing diets. The enzyme composition and declared activities of the premix are provided in Table S1. In this study, MLE refers to diets containing sun-dried mulberry leaf powder together with a multi-enzyme premix.

Based on the essential amino acid (EAA) requirements of white sturgeon [33] and a previous study [34], crystalline amino acids (L-Lys-HCl, DL-Met, and L-Thr), fish oil, and monocalcium phosphate were incorporated to balance dietary EAA, essential fatty acids and available phosphorus, respectively. The formulation and proximate composition of each diet are presented in Table S2.

A total of 1000 *Acipenser schrenckii* juveniles were obtained from Beijing Tianyuanyugang Farm Co., Ltd. (Beijing Amur Sturgeon Elite Breeding Base, Beijing, China). The experimental fish were sexually undifferentiated individuals with no genetic modification and had not received any experimental treatments before this trial. Prior to the feeding trial, 320 fish with an initial body weight of 199.16 ± 1.42 g were acclimatized to experimental conditions for 4 weeks and fasted for 24 h. The experimental fish were randomly selected from the holding pool and allocated into 16 net cages (1 × 1 × 1.5 m), with 20 fish per cage and four replicates per dietary treatment (four treatments × four replicates; 320 fish in total). All net cages were evenly suspended in the pond to balance variations in light exposure, water flow and water quality. Fish were fed twice daily for 10 weeks. On sampling days, the sampling sequence of net cages was randomized to reduce systematic bias arising from measurement order.

At the fish allocation stage and during daily feeding and routine management, the researchers responsible for husbandry knew the group allocation, as the experimental diets differed visually, making blinding impractical during feeding. During sampling, tissue dissection and laboratory measurement, all samples were labeled only with serial numbers without treatment information. Operators performing the laboratory analyses were unaware of group identity. Group information was unblinded only after all measurements were completed, prior to formal data analysis. Water quality parameters were maintained as follows: temperature 20 ± 2 °C, pH 7.5–8.5, and dissolved oxygen > 7.0 mg/L.

### 2.3. Sample and Data Collection

Feed consumption, mortality and body weight were recorded daily throughout the trial. At the end of the feeding period, fish were fasted for 24 h before sampling. Twelve fish per treatment were randomly captured and anesthetized with MS-222 (100 mg/L). Final body weight, body length, liver weight and visceral weight were measured to calculate growth indices. Blood samples were collected from the caudal vein, allowed to stand at room temperature for 30 min, and subsequently centrifuged at 4 °C to harvest serum for biochemical analysis.

Foregut tissue was rapidly frozen in liquid nitrogen and stored at −80 °C for the determination of intestinal digestive enzyme activities. Approximately 2 cm foregut segments were fixed in 4% paraformaldehyde for histological observation. Around 0.5 g of midgut and hindgut tissues were immersed in RNAlater (Beyotime, Shanghai, China), incubated overnight at 4 °C, and preserved at −80 °C for RNA extraction. For multi-omics analysis, paired intestinal samples were collected from the same six individual fish. Briefly, intestinal tissue without digesta was snap-frozen in liquid nitrogen for untargeted metabolomics analysis, and intestinal contents from the same corresponding individuals were collected and preserved under identical conditions for gut microbiota sequencing. This paired sampling strategy ensured that the metabolomic and microbiota data were matched at the individual fish level, allowing individual-level correlation analysis.

### 2.4. Growth Performance

The weight gain rate (WGR), specific growth rate (SGR), feed conversion ratio (FCR), feeding rate (FR), survival rate (SR), condition factor (CF), hepatosomatic index (HSI), and viscerosomatic index (VSI) were calculated using the following formulas:

WGR (%) = 100 × (Wt − Wo)/Wo

SGR (%/d) = 100 × [ln(Wt) − ln(Wo)]/days

FCR = feed intake/(Wt − Wo + weight of dead fish)

FR (%) = 100 × total dry feed intake/[(Wt + Wo)/2]/days

SR (%) = 100 × final number of fish/initial number of fish

CF (g/cm^3^) = 100 × body weight/(body length)^3^

HSI (%) = 100 × liver weight/body weight

VSI (%) = 100 × visceral weight/body weight

where Wo = initial body weight; Wt = final body weight.

### 2.5. Biochemistry Indices

Serum triglyceride (TG, Cat. No. TG6160), total cholesterol (TC, Cat. No. TC6150), glucose (GLU, Cat. No. GL6210), immunoglobulin M (IgM, Cat. No. IG7437), complement 3 (C3, Cat. No. C37392), and complement 4 (C4, Cat. No. C47402) were determined using commercial assay kits from Beijing Leadman Biochemical Co., Ltd., Beijing, China. Serum total antioxidant capacity (T-AOC, Cat. No. A015-2), superoxide dismutase (SOD, Cat. No. A001-1), malondialdehyde (MDA, Cat. No. A003-1), as well as intestinal amylase (AMS, Cat. No. C016-1-1), lipase (LPS, Cat. No. A054-2-1) and trypsin (Cat. No. A080-2-1) were measured with kits supplied by Nanjing Jiancheng Bioengineering Institute, Nanjing, China.

### 2.6. Intestinal Histology

After fixation in 4% paraformaldehyde, intestinal tissues were subjected to dehydration, embedding, sectioning and hematoxylin and eosin (H&E) staining. Villus height and crypt depth were visualized using a Nikon Eclipse E100 microscope (Nikon, Tokyo, Japan) and quantified with Image-Pro Plus 6.0 software (Media Cybernetics, Rockville, MD, USA).

### 2.7. Intestinal Microbiota Diversity Analysis

Total genomic DNA was extracted from intestinal tissues and digesta. Paired-end sequencing of bacterial DNA fragments was performed on an Illumina NovaSeq platform (Illumina, Inc., San Diego, CA, USA). DNA concentration and quality were verified using a NanoDrop NC2000 spectrophotometer (Thermo Fisher Scientific, Waltham, MA, USA) and agarose gel electrophoresis, respectively. The V3–V4 region of the bacterial 16S rRNA gene was amplified using forward primer 338F and reverse primer 806R. PCR amplicons were purified using Vazyme VAHTSTM DNA Clean Beads (Vazyme, Nanjing, China) and quantified with the Quant-iT PicoGreen dsDNA Assay Kit (Invitrogen, Carlsbad, CA, USA). Amplicons were pooled in equal amounts and sequenced (2 × 250 bp) using the NovaSeq 6000 SP Reagent Kit (500 cycles) at Shanghai Personal Biotechnology Co., Ltd., Shanghai, China. After quality filtering, an average of approximately 50,000 tags per sample was retained for downstream analysis.

Primer sequences were removed using the cutadapt plugin in QIIME2, and reads without matched primers were discarded. Sequence denoising and amplicon sequence variant (ASV) clustering were then conducted using the QIIME2 dada2 denoise-paired pipeline, including quality filtering, denoising, merging, and chimera removal, together with VSEARCH [35,36]. Taxonomic classification of ASVs was performed using the Greengenes2 database. Alpha diversity metrics including Chao1, observed species, Shannon and Simpson indices were calculated at the ASV level. Non-metric multidimensional scaling (NMDS) was performed to visualize differences in microbial community structure among treatments. A permutation test of multivariate homogeneity of group dispersions (PERMDISP) and Adonis analysis were used to test intergroup differences. Differential taxa were screened via Kruskal–Wallis and Wilcoxon tests. Linear discriminant analysis effect size (LEfSe) was applied to identify biomarker taxa with thresholds set as LDA score > 2 and *p* < 0.05.

### 2.8. Untargeted Metabolomics Analysis Based on LC-MS/MS

Briefly, 30 mg of intestinal tissue was homogenized twice (55 Hz, 60 s) with steel beads in pre-cooled water. Methanol–acetonitrile (1:1, *v*/*v*) was added, followed by ultrasonication for 30 min, incubation at −20 °C for 45 min, and centrifugation at 12,000 rpm (4 °C, 20 min). The supernatant was vacuum-dried, reconstituted in 50% methanol containing 5 ppm 2-chlorophenylalanine, vortexed and centrifuged. The supernatant was filtered through a 0.22 μm membrane and transferred to sample vials.

Metabolite profiling was carried out using a Vanquish UHPLC system coupled with an Orbitrap Exploris 120 mass spectrometer (Thermo Fisher Scientific, Waltham, MA, USA) equipped with an electrospray ionization source. Chromatographic separation was performed on an ACQUITY UPLC HSS T3 column (100 Å, 1.8 μm, 2.1 mm × 100 mm; Waters, Milford, MA, USA) at 40 °C. The mobile phase consisted of (A) 0.1% formic acid in water and (B) acetonitrile containing 0.1% formic acid, and the same mobile phase was used for both positive and negative ionization modes. The gradient program was as follows: 0–1 min, 5% B; 1–4.7 min, 5–95% B; 4.7–6 min, 95% B; 6–6.1 min, 95–5% B; 6.1–8.5 min, 5% B. The flow rate was 0.4 mL/min, the autosampler temperature was 8 °C, and the injection volume was 2 μL. Metabolites were detected in positive (POS) and negative (NEG) ionization modes. Pooled quality-control (QC) samples were prepared by mixing equal aliquots from all samples and were injected at regular intervals throughout the analytical run to monitor system stability and data quality. The injection order of all samples was randomized.

Raw data (.raw) were imported into MS-DIAL (version 4.9.221218) for peak extraction and alignment. Missing values were filled using K-nearest neighbor (KNN) imputation, and the data were normalized by total peak area. Features with a QC relative standard deviation (RSD) > 0.3 were excluded from downstream analysis. Orthogonal partial least squares discriminant analysis (OPLS-DA) was applied to discriminate metabolic profiles among treatments. Differential metabolites were screened according to variable importance in projection (VIP) > 1, *p* < 0.05 and fold change (FC) > 1. Metabolite identification confidence levels were assigned according to the Metabolomics Standards Initiative (MSI) framework, and only metabolites with identification confidence level 2 or above were retained for downstream analysis. Metabolites obtained from positive and negative modes were merged, and duplicates were removed by retaining compounds with higher VIP values.

Metabolite annotation was performed against the KEGG species database. Because KEGG annotations for *Acipenser schrenckii* are currently unavailable, *Acipenser ruthenus*, a closely related species within the same genus, was used as the closest available reference for KEGG pathway annotation. Fisher’s exact test was then used to determine significantly enriched metabolic pathways.

### 2.9. Correlation Analysis Between Intestinal Microbiota and Metabolites

Spearman’s rank correlation coefficients were calculated between genus-level differential microbiota and differential metabolites to assess potential associations. Correlation pairs satisfying *p* < 0.05 and |correlation coefficient| > 0.5 were retained. The top 20 microorganisms and metabolites ranked by correlation magnitude were selected to construct clustered correlation heatmaps. Red blocks indicate significant positive correlations, blue-purple blocks represent significant negative correlations, and asterisks denote statistically significant correlations. These analyses indicate statistical associations and do not establish causal relationships. The integrated interpretation of microbiota and metabolomics data should therefore be performed cautiously, as multi-omics approaches can provide valuable insights into diet-related host–microbiota interactions while requiring appropriate statistical validation [37].

### 2.10. RNA Isolation, Reverse Transcription, and RT-qPCR Analysis

Total intestinal RNA was extracted using the RNA EasyFast Kit (Tiangen Biochemical Technology Co., Beijing, China). RNA integrity was verified via 1% agarose gel electrophoresis, and RNA concentration and purity (OD260/280) were determined using a NanoDrop 2000 spectrophotometer (Thermo Fisher Scientific, Waltham, MA, USA).

β-actin (GenBank accession no. AY649619) and gene-specific primers for *Acipenser schrenckii* were adopted from a previous study [38], as listed in Table S3. RT-qPCR was performed on a qTOWER Iris instrument (Analytik Jena, Jena, Germany) using TB Green^®^ Premix Ex Taq™ II (Takara, Tokyo, Japan), with three technical replicates per sample. The thermal profile consisted of an initial denaturation at 95 °C for 30 s, followed by 40 cycles of 95 °C for 5 s and 60 °C for 34 s. Relative gene expression was quantified using the 2^−ΔΔCt^ method.

### 2.11. Statistical Analysis

Conventional phenotypic data, including growth performance, serum biochemical indices, intestinal digestive enzyme activities, intestinal histomorphology and gene expression, were analyzed using GraphPad Prism 10.6.0 (San Diego, CA, USA). Tests for normality and homogeneity of variance were conducted prior to parametric analysis. One-way analysis of variance (ANOVA) followed by Tukey’s multiple comparisons test was used to compare differences among dietary treatments; means were labeled with different superscript letters where appropriate. When parametric assumptions were violated, non-parametric statistical methods were adopted. Linear and second-order polynomial (quadratic) regression analyses were conducted to evaluate dose–response relationships between dietary MLE levels and all measured variables.

Intestinal microbiota, untargeted metabolomics and microbiota–metabolite correlation analyses described in Section 2.7, Section 2.8 and Section 2.9 were performed using R software (v4.2.1). Statistical significance was defined as *p* < 0.05 for all analyses, and data are expressed as mean ± standard error of the mean (SEM).

## 3. Results

### 3.1. Growth Performance and Morphometric Parameters

Compared with the CK group, MLE6 significantly reduced FBW, WGR and SGR, and increased FCR (*p* < 0.05), and these growth parameters exhibited significant linear trends. No significant differences in FR, CF, HSI and VSI were detected among the treatments (*p* > 0.05) (Table 1).

### 3.2. Serum Biochemical, Antioxidant, and Immune Indices

The serum biochemical, antioxidant, and immune indices are presented in Table 2. Compared with the CK group, serum TC, TG and GLU concentrations were significantly decreased (*p* < 0.05). Quadratic responses were observed for TC and TG, with the minimum values estimated at dietary MLE inclusion levels of 3.75% and 3.82%, respectively.

For antioxidant indices, dietary MLE supplementation significantly increased serum T-AOC and T-SOD activities in the treatment groups. T-SOD exhibited a significant linear increasing trend, while T-AOC showed a quadratic response, with its maximum value estimated at the MLE inclusion level of 4.01%. Serum MDA content was significantly reduced in the MLE4 and MLE6 groups (*p* < 0.05) and displayed a quadratic pattern, with the minimum value estimated at 5.20% MLE supplementation.

In terms of immune parameters, serum C3, C4 and IgM levels were markedly elevated in all MLE treatments relative to the CK group. All three immune indicators showed significant quadratic responses (first rising and then declining), and their maximum values were estimated at MLE inclusion levels of 3.90%, 4.91% and 4.03%, respectively.

### 3.3. Changes in Intestinal Digestive Function and Morphology

**Figure 1:**
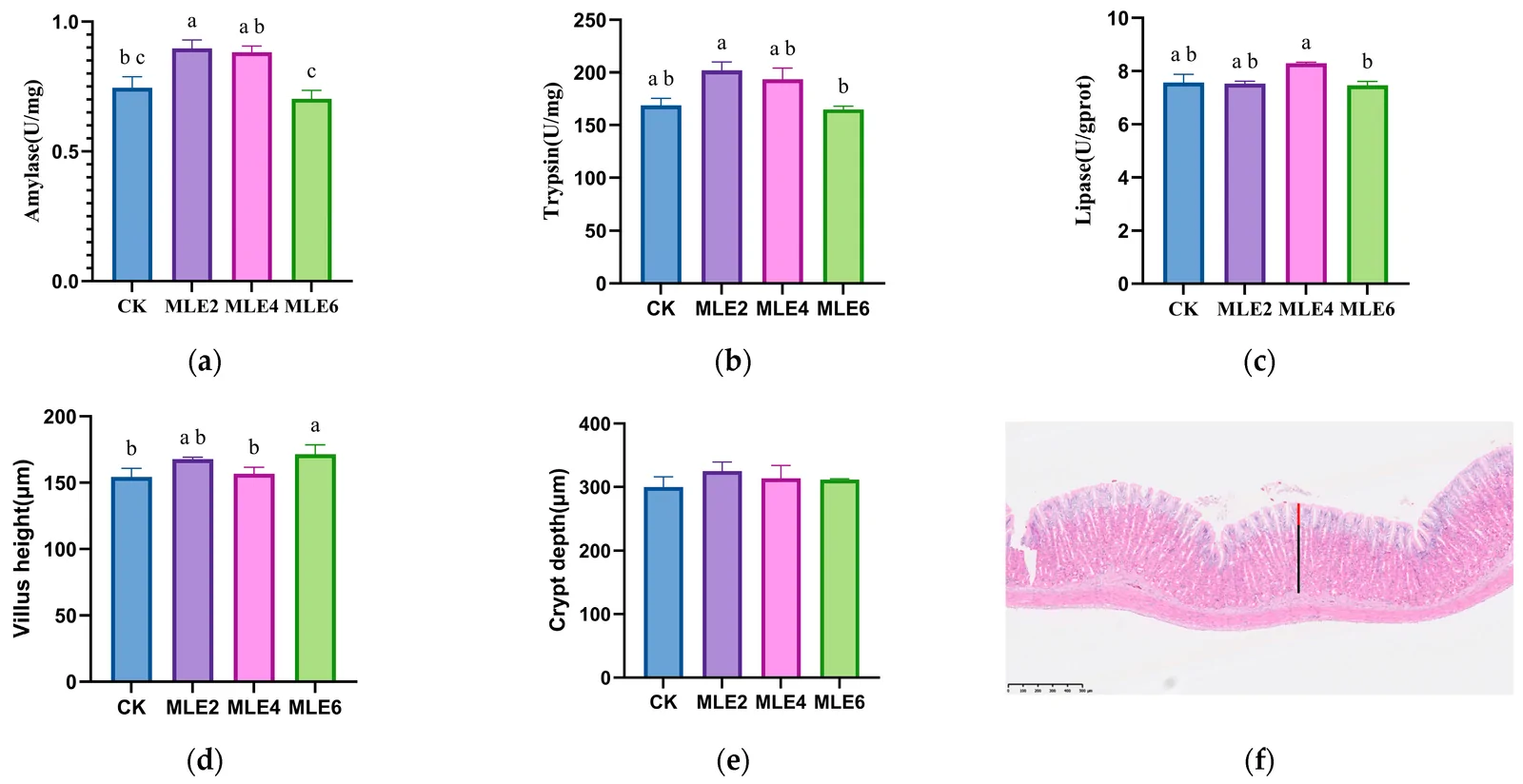
Effects of dietary MLE supplementation on intestinal digestive enzymes and morphology of juvenile *Acipenser schrenckii* (*n* = 6). (**a**–**c**) Intestinal digestive enzymes: (**a**) amylase activity; (**b**) trypsin activity; (**c**) lipase activity. (**d**–**f**) Intestinal morphological indices: (**d**) villus height; (**e**) crypt depth; (**f**) representative H&E histological micrographs of intestinal tissue. Red lines indicate villus height, and black lines indicate crypt depth. In (**a**–**e**), blue bars represent the CK group, purple bars represent the MLE2 group, pink bars represent the MLE4 group, and green bars represent the MLE6 group. Data are presented as mean ± SEM. Values with different lowercase letters denote significant differences among treatments (*p* < 0.05).

Compared with the CK group, amylase activity was significantly higher in the MLE2 group (Figure 1a, *p* < 0.05). No significant differences in lipase and trypsin activities were detected among treatments (Figure 1b,c, *p* > 0.05), although increasing trends were observed in the MLE2 and MLE4 groups. Villus height was significantly higher in the MLE6 group (Figure 1d, *p* < 0.05). Crypt depth did not differ significantly across the groups (Figure 1e, *p* > 0.05).

### 3.4. Intestinal Microbial Community Composition and Diversity of Acipenser schrenckii

#### 3.4.1. Alpha and Beta Diversity Analysis

**Figure 2:**
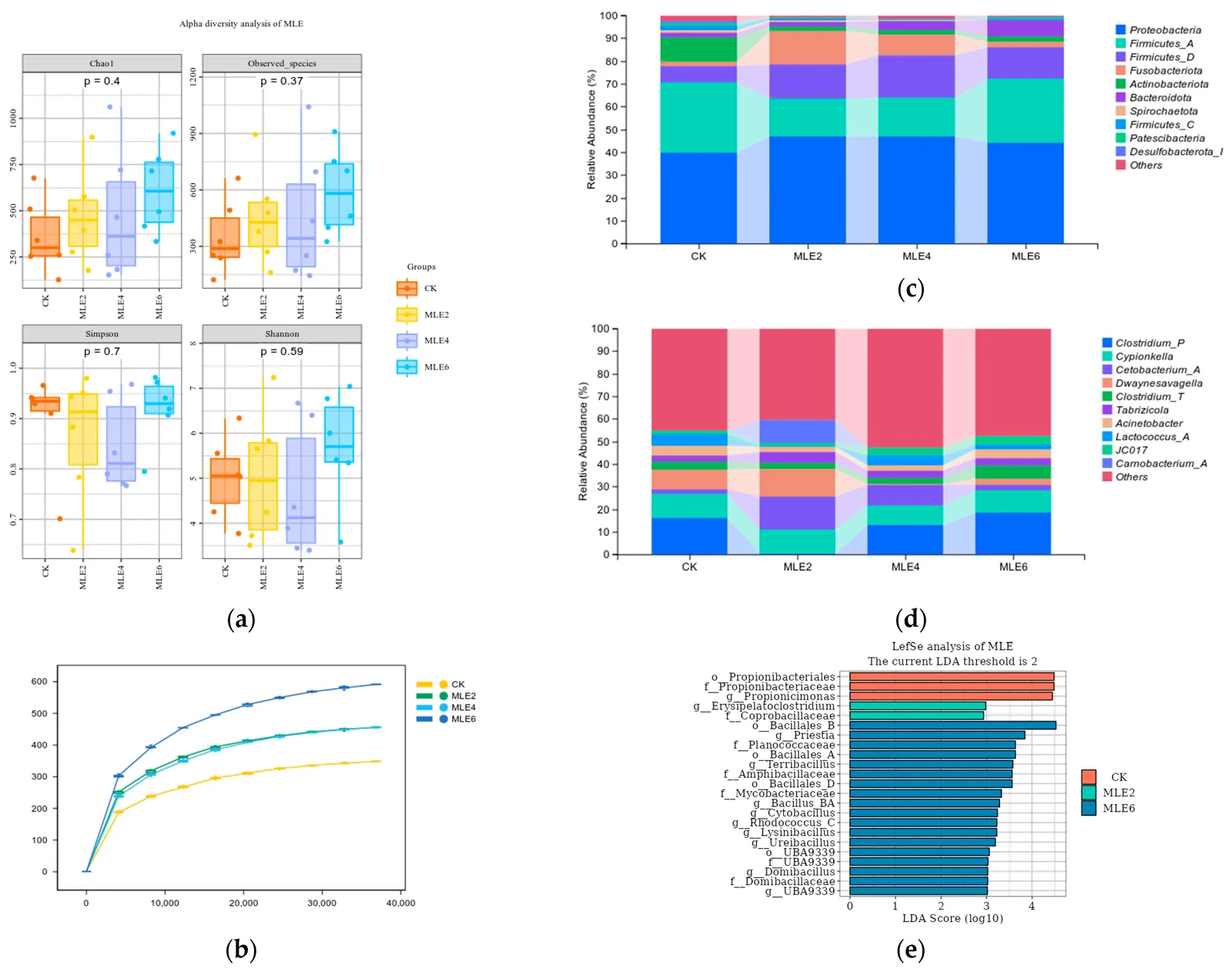
Effects of dietary MLE supplementation on intestinal microbial diversity and community composition of juvenile *Acipenser schrenckii* (*n* = 6). (**a**) Alpha diversity indices of intestinal microbiota among different groups. (**b**) Rarefaction curves. (**c**) Microbial community composition at the phylum level. (**d**) Microbial community composition at the genus level. (**e**) Group-specific differential microbial biomarkers identified via LEfSe analysis.

No significant differences in alpha diversity indices were observed between the MLE-supplemented groups and the CK group (*p* > 0.05) (Figure 2a). The rarefaction curves tended to plateau, indicating that the sequencing depth was sufficient to capture the microbial diversity of all samples (Figure 2b). The stress value of the NMDS ordination was below 0.2 (stress = 0.138), supporting the reliability of the ordination. No significant intergroup differences were found in overall microbial community structure (Adonis, *p* = 0.228) or community dispersion (PERMDISP, *p* = 0.339), and no significant differences were detected in any pairwise comparisons (Table S4).

#### 3.4.2. Species Composition Analysis

The top 10 dominant phyla showed that Proteobacteria, Firmicutes (including Firmicutes_A and Firmicutes_D), Fusobacteriota, and Actinobacteriota dominated the intestinal microbiota across all groups. Among these phyla, Actinobacteriota was more abundant in the CK group, whereas Fusobacteriota was more abundant in the MLE2 and MLE4 groups. In addition, Firmicutes_D was more abundant in all MLE treatment groups, while Firmicutes_A was more abundant in the CK and MLE6 groups (Figure 2c). At the genus level, *Clostridium_P* was more abundant in CK, MLE2, and MLE6; *Cetobacterium_A* was enriched in MLE2 and MLE4; and *Dwaynesavagella* was more abundant in CK and MLE2 (Figure 2d).

#### 3.4.3. Identification of Potential Intestinal Microbial Biomarkers

LEfSe analysis showed that taxa enriched in CK (Propionibacteriales, Propionibacteriaceae, and *Propionicimonas*) belonged to Actinobacteriota. *Erysipelatoclostridium* and Coprobacillaceae (Firmicutes_D) served as biomarker taxa for MLE2. The MLE6 group had the greatest number of differential taxa, mainly assigned to Firmicutes_D, including multiple Bacillales orders and Bacillaceae genera such as *Priestia*, *Terribacillus*, *Bacillus_BA*, and *Lysinibacillus* (Figure 2e).

### 3.5. Untargeted Metabolomics Analysis of Differential Metabolites

Model parameters indicated satisfactory fitness and predictive capacity of the OPLS-DA model (Table S5). Permutation test results showed gradual decreases in R^2^ and Q^2^ as the retention degree declined, accompanied by an upward regression trend (Figure S1). These results indicated no obvious overfitting and supported the reliability of the constructed model [39].

#### 3.5.1. Screening for Differential Metabolites

**Figure 3:**
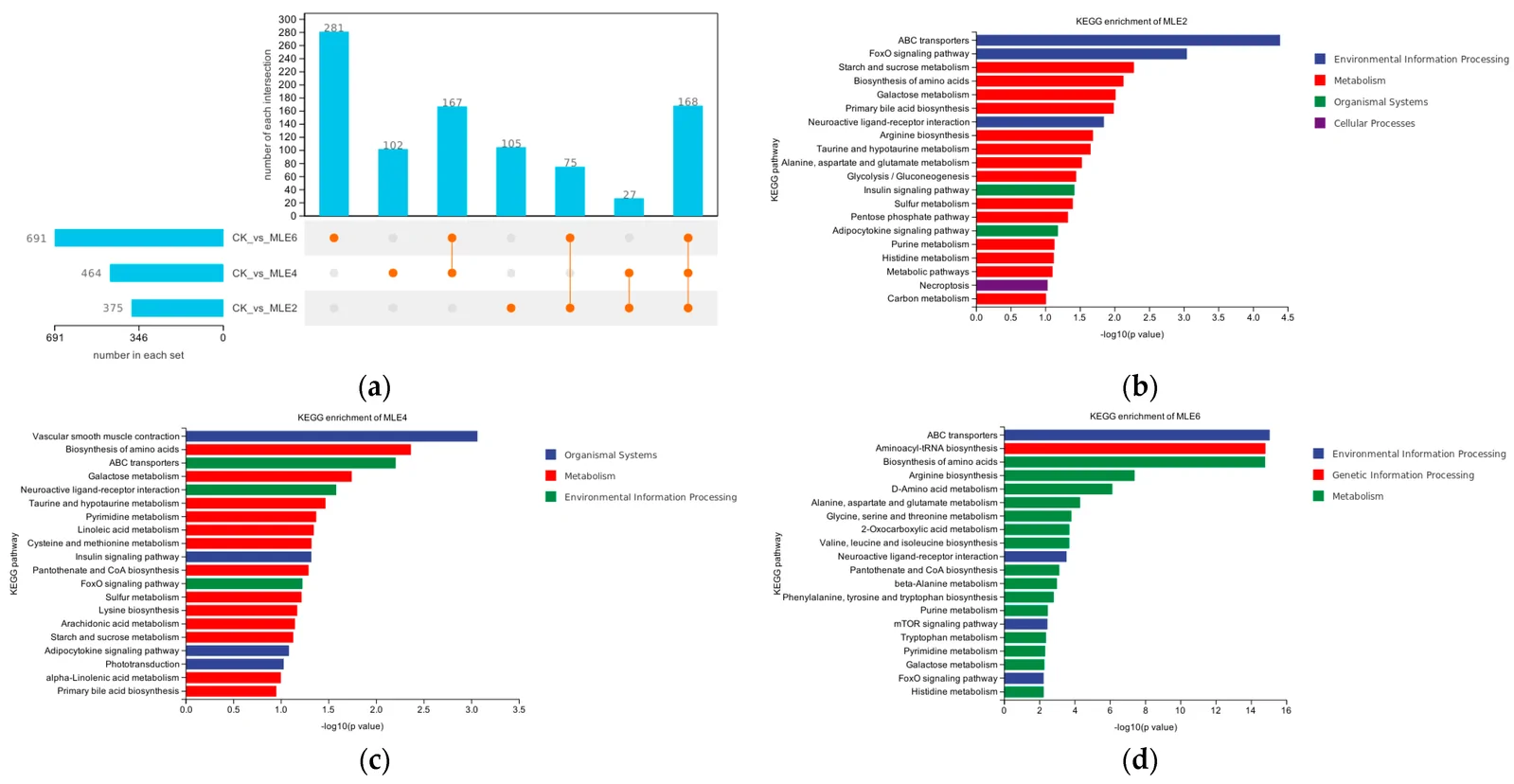
Distribution of differential metabolites and KEGG pathway enrichment analysis (*n* = 6). (**a**) UpSet plot illustrating shared and unique differential metabolites across comparison groups. (**b**–**d**) KEGG pathway enrichment analysis of differential metabolites from the comparisons of CK vs. MLE2 (**b**), CK vs. MLE4 (**c**), and CK vs. MLE6 (**d**), respectively. The x-axis represents −log_10_ (*p*-value), and the y-axis represents enriched KEGG pathways.

Compared with the CK group, 375, 464, and 691 differential metabolites were identified in the MLE2, MLE4, and MLE6 groups, respectively. The numbers of uniquely altered metabolites were 105, 102, and 281, while 168 metabolites exhibited shared differential abundance across all MLE treatments (Figure 3a). Sixty-three metabolites were consistently downregulated, including one alkaloid and derivative, six benzenoids, 12 lipids and lipid-like molecules, three nucleosides, nucleotides and analogs, 13 organic acids and derivatives, three organic oxygen compounds, seven organoheterocyclic compounds, and three phenylpropanoids and polyketides. Ninety-nine metabolites were consistently upregulated, covering one alkaloid and derivative, four benzenoids, 16 lipids and lipid-like molecules, 17 organic acids and derivatives, 10 organic oxygen compounds, nine organoheterocyclic compounds, and seven phenylpropanoids and polyketides.

#### 3.5.2. KEGG Pathway Enrichment Analysis

To further explore the biological functions of differential metabolites, KEGG pathway enrichment analysis was conducted for each MLE treatment.

In the MLE2 group, ABC transporters, starch and sucrose metabolism, and primary bile acid biosynthesis were the most enriched pathways (Figure 3b). For MLE4, vascular smooth muscle contraction, biosynthesis of amino acids, and ABC transporters were the top enriched pathways; enrichment was also observed in lipid metabolism (linoleic acid and arachidonic acid metabolism) and multiple signal transduction pathways (Figure 3c). In the MLE6 group, ABC transporters, aminoacyl-tRNA biosynthesis, and biosynthesis of amino acids dominated the enrichment profiles, together with multiple amino acid-related pathways and enrichment of purine metabolism. Moreover, the mTOR and FoxO signaling pathways were specifically enriched in the MLE6 group (Figure 3d).

### 3.6. Correlation Analysis Between Microorganisms and Metabolites

All differential metabolites identified across treatments were clustered into four distinct modules (M-C1 to M-C4), and each module contained metabolite sets with divergent abundance patterns among groups.

**Figure 4:**
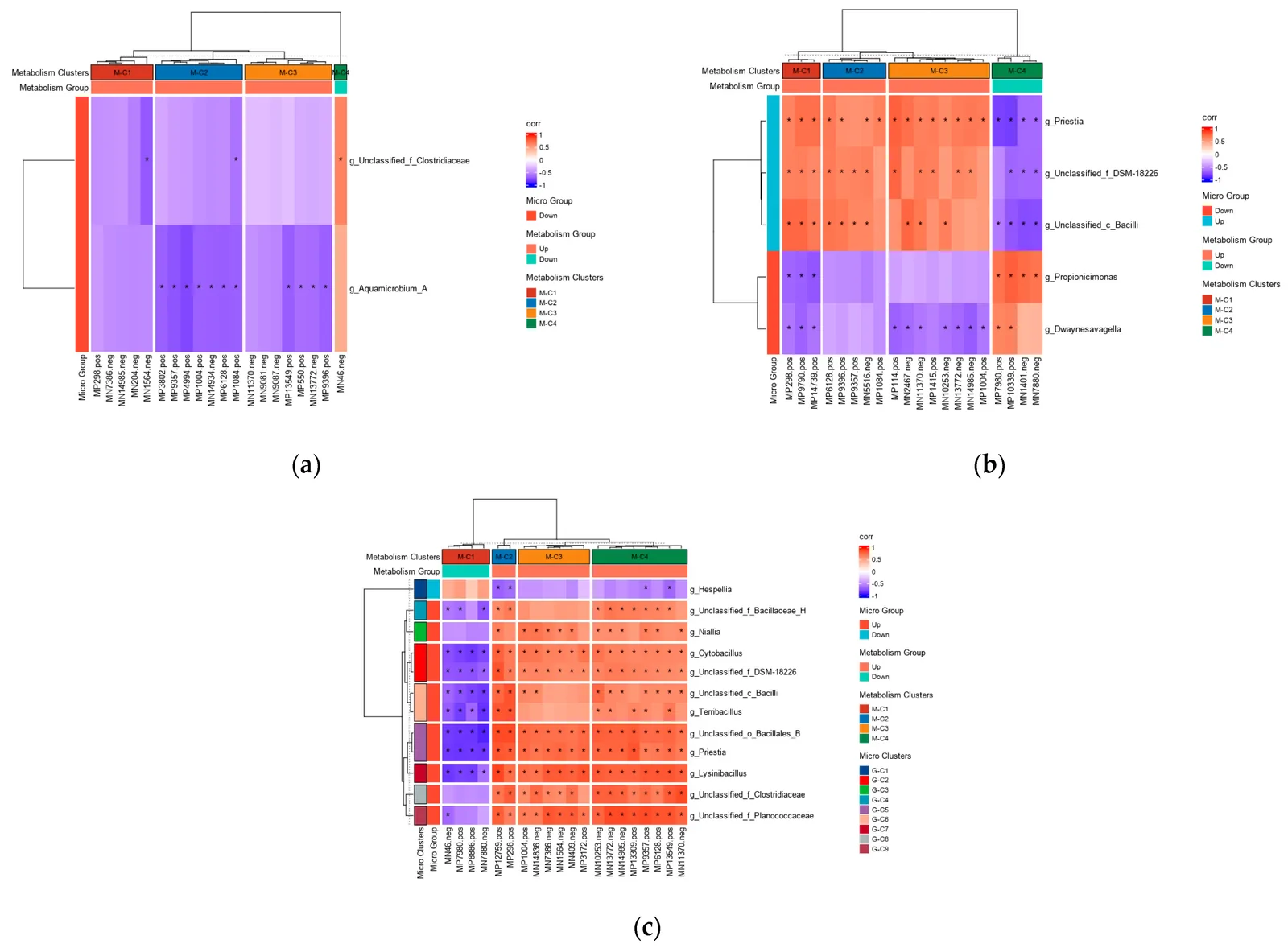
Correlation heatmap of differential intestinal microbial genera and metabolites under graded dietary MLE supplementation (*n* = 6). (**a**) MLE2 vs. CK; (**b**) MLE4 vs. CK; (**c**) MLE6 vs. CK. The horizontal axis shows differential intestinal microbial genera, and the vertical axis represents differential metabolites. Red indicates significant positive correlations, whereas blue-purple indicates significant negative correlations. Asterisks denote statistically significant correlations (*p* < 0.05).

In the MLE2 group, microbial taxa enriched in MLE2 were negatively correlated with upregulated metabolites (Figure 4a). The number and strength of positive correlations between intestinal microbiota and elevated metabolites increased with increasing dietary MLE inclusion. The MLE4 group harbored enriched taxa including *Priestia*, unclassified_f_DSM-18226 and unclassified_c_Bacilli; these genera were positively correlated with MLE4-specific upregulated metabolites and negatively correlated with the downregulated module M-C4 (Figure 4b). In the MLE6 group, multiple *Bacillus*-related genera showed positive correlations with upregulated modules M-C2 to M-C4. In contrast, only the depleted taxon *Hespellia* displayed significant negative correlations with upregulated metabolic modules (Figure 4c). These correlation results are exploratory and indicate statistical association rather than causal relationships.

### 3.7. RNA Isolation, Reverse Transcription, and mRNA Analysis

#### 3.7.1. Antioxidant-Related Gene Expression

**Figure 5:**
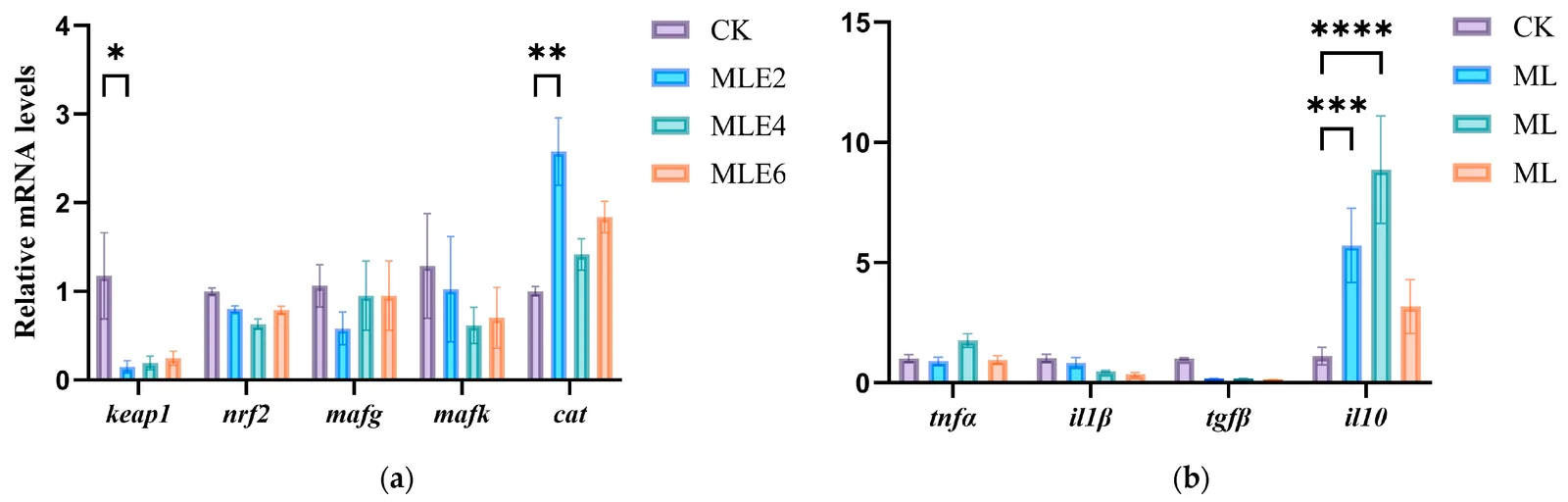
Effects of dietary MLE supplementation on antioxidant and immune-related gene expression in juvenile *Acipenser schrenckii* (*n* = 3). (**a**) Antioxidant-related genes (*keap1*, *nrf2*, *mafg*, *mafk*, *cat*); (**b**) Immune and inflammatory cytokine genes (*tnfα*, *il1β*, *tgfβ*, *il10*). Data are presented as mean ± SEM; error bars represent SEM. *, **, ***, and **** denote significant differences at *p* < 0.05, *p* < 0.01, *p* < 0.001, and *p* < 0.0001, respectively.

The transcriptional levels of core genes in the Nrf2/Keap1 antioxidant pathway were measured (Figure 5a). Compared with the CK group, *keap1* mRNA expression was downregulated in all MLE treatments, with a significant decrease observed in the MLE2 group (*p* < 0.05). The transcription factor gene *nrf2* and its cofactor genes *mafg* and *mafk* showed mild but non-significant downregulation in the treatment groups (*p* > 0.05). In contrast, *cat* was significantly upregulated in the MLE2 group (*p* < 0.05).

#### 3.7.2. Immune-Related Gene Expression

Intestinal immune and inflammatory gene expression was further analyzed (Figure 5b). The pro-inflammatory genes *tnfa* and *il1β*, as well as the immunoregulatory gene *tgfβ*, showed no significant differences among treatments. However, the anti-inflammatory cytokine gene *il10* was significantly upregulated in the MLE2 and MLE4 groups (*p* < 0.05), with the highest expression level observed in the MLE4 group (*p* < 0.0001).

## 4. Discussion

Mulberry leaves are rich in bioactive components such as polyphenols and 1-deoxynojirimycin (DNJ), and enzymatic hydrolysis can further improve the bioavailability of these compounds, making mulberry leaf powder supplemented with a multi-enzyme premix (MLE) a promising functional feed ingredient for aquaculture. In the present study, we systematically investigated the effects of graded dietary MLE supplementation on growth performance, serum glycolipid metabolism, intestinal morphology, digestive enzyme activity, antioxidant capacity, immune response, intestinal microbiota and metabolome of juvenile *Acipenser schrenckii*. Collectively, our results indicate that appropriate MLE inclusion was associated with improved intestinal physiological homeostasis through a coordinated cascade involving taxon-specific changes in gut microbiota, altered metabolic profiles, and changes in Nrf2/Keap1-related antioxidant indicators, whereas excessive MLE supplementation was associated with adverse effects on fish growth in a dose-dependent manner. These findings provide a basis for linking dietary MLE to intestinal health and identify a preliminary optimal dosage range that requires validation in future dose–response studies before practical application in sturgeon cultivation.

### 4.1. Growth Performance, Serum Glycolipid Metabolism, and Intestinal Digestive and Morphological Responses to Graded MLE Supplementation

Growth performance exhibited a dose-dependent response to dietary MLE supplementation. Relative to the control, neither 2% nor 4% MLE significantly improved growth, and final body weight, weight gain rate (WGR), and specific growth rate (SGR) showed a numerical decline as MLE inclusion increased, with a significant negative linear trend. Moderate MLE inclusion (2–4%) maintained normal growth of juvenile *Acipenser schrenckii*, whereas high-dose MLE (6%) significantly impaired growth. This adverse outcome is likely related to excessive crude fiber and accumulated anti-nutritional factors in mulberry leaves at high inclusion levels [40]. Notably, processing methods greatly modify the nutritional value of mulberry leaf products; previous studies revealed that differently processed mulberry materials exert divergent effects on fish growth and metabolism [41,42]. Importantly, compared with studies using raw mulberry leaf meal, the multi-enzyme premix applied in our study likely facilitated the release of bound polyphenols and 1-deoxynojirimycin (DNJ) from the plant cell wall matrix, which may be associated with the physiological benefits in antioxidant capacity, immune-related parameters, and intestinal health observed at lower inclusion rates (2–4%) without a growth penalty. This highlights that supplementing mulberry leaf with a multi-enzyme premix may be an effective strategy to mitigate the adverse effects of crude fiber and anti-nutritional factors while maximizing the bioavailability of functional components. The combined application of mulberry leaf and exogenous enzymes can also alleviate growth limitations to some extent [27]. Therefore, screening an appropriate MLE dosage is critical to avoid negative impacts on fish production, and the present results suggest that the dosage associated with physiological benefits (2–4%) should be distinguished from the dosage that maximizes growth, which in this study remained the control diet.

Beyond growth indices, graded MLE supplementation was associated with changes in serum glycolipid homeostasis. The decreased serum triglyceride, total cholesterol, and glucose concentrations in MLE groups indicated that bioactive ingredients in mulberry leaf powder supplemented with a multi-enzyme premix, including polyphenols and DNJ, may participate in modulating lipid synthesis and carbohydrate metabolism [43]. Similar hypoglycaemic and lipid-regulating effects of mulberry leaf extracts have been documented in mandarin fish and Chinese giant salamander [44]. Regression analysis based on multiple physiological indicators further provided a preliminary estimate of the optimal inclusion range of MLE, which requires validation in future dose–response studies before practical application.

Intestinal digestive capacity and morphological integrity are fundamental to efficient nutrient utilization [45]. In the present study, amylase activity was significantly elevated in the MLE2 group, suggesting that appropriate MLE supplementation may be associated with improved carbohydrate digestion. Moreover, increased intestinal villus height in MLE-treated groups expanded the absorption area and may help sustain intestinal epithelial barrier function [46,47]. Comparable improvements in digestive enzyme activity and intestinal morphology under suitable mulberry leaf supplementation were also observed in mandarin fish [43]. Collectively, moderate MLE inclusion was associated with improved intestinal digestive function and morphological structure, and may facilitate nutrient utilization. However, such beneficial effects diminished under excessive MLE addition. Notably, the impaired intestinal morphology and reduced digestive enzyme activities in the MLE6 group were closely associated with the diminished antioxidant capacity (Section 4.2) and disrupted inflammatory homeostasis (Section 4.3) observed at the same dosage. These findings suggest that excessive MLE-induced oxidative stress and mild inflammation in the gut epithelium may have contributed to the observed growth retardation, although nutrient dilution or fiber overload cannot be excluded. This potential linkage may help explain why the MLE6 group exhibited the most pronounced growth depression despite receiving the highest concentration of bioactive compounds.

### 4.2. Effects of MLE on Intestinal Antioxidant Capacity and Nrf2/Keap1 Signaling Pathway

Mulberry leaves are rich in bioactive polysaccharides and flavonoids, which exhibit potent free radical scavenging and antioxidant properties [48,49]. Serum T-AOC, SOD, and MDA are classic and reliable biomarkers for assessing systemic antioxidant defense capacity and oxidative stress status in aquatic animals [50]. In the present study, graded dietary MLE supplementation exerted significant dose-dependent antioxidant regulation in juvenile *Acipenser schrenckii*, showing typical quadratic response characteristics. The optimal antioxidant response was observed at moderate MLE inclusion levels, with the maximum T-AOC value at 4.01% and the minimum MDA concentration at 5.20%. These quadratic trends indicated that appropriate MLE supplementation was associated with enhanced systemic antioxidant capacity, whereas excessive MLE inclusion was associated with diminished antioxidant benefits and may increase oxidative stress accumulation.

At the molecular level, the Keap1-Nrf2 signaling pathway serves as a core regulatory axis of cellular antioxidant responses in fish [51]. In the present study, *keap1* was significantly downregulated only in the MLE2 group, whereas no significant differences in the transcript levels of *nrf2*, *mafg*, and *mafk* were observed across treatments. It should be noted that Nrf2/Keap1 signaling is also regulated at the post-translational level (protein stabilization and nuclear translocation), so transcript abundance of *nrf2* alone may not fully reflect pathway activity. Consistently, the antioxidant enzyme gene *cat* was significantly upregulated in the low-dose MLE2 group, implying enhanced downstream antioxidant enzyme responses under low-dose MLE supplementation. The elevation of serum T-AOC and SOD together with reduced MDA in moderate MLE groups supported improved systemic antioxidant status, which may be partially associated with repression of *keap1* and changes in downstream antioxidant indicators [52]. Similar antioxidant modulation induced by mulberry leaf products has been documented in multiple aquatic species [18,44,46]. Plant-derived polyphenols and polysaccharides generally exhibit prominent free radical scavenging capacity and immunomodulatory effects in aquaculture [53]. Notably, the physiological effects of mulberry leaf materials rely heavily on processing technologies [41].

The attenuation of antioxidant benefits at high MLE inclusion (6%) may be further explained by the hormetic characteristics of dietary polyphenols. At moderate doses, polyphenols can trigger antioxidant protective responses, partly linked to Keap1 repression and downstream antioxidant enzyme induction [54]. Similarly, mulberry leaf polyphenols have been demonstrated to beneficially modulate intestinal flora and lipid metabolism in murine models at appropriate dosages [55]. However, at supra-optimal doses, the overwhelming polyphenol load may exceed the capacity of the endogenous antioxidant defense system, shifting the redox balance towards a pro-oxidant state and thereby counteracting the initial antioxidant benefits. This hormetic model provides a conceptual framework for understanding the quadratic dose–response curves observed across multiple physiological indices in our study. Collectively, low-dose dietary MLE suppressed *keap1* transcription and upregulated *cat*, which may contribute to the enhanced antioxidant capacity of sturgeon. Nevertheless, the consistent quadratic dose effects indicated that high-dose MLE supplementation exceeded the optimal physiological regulatory range, resulting in attenuated antioxidant benefits and progressive oxidative damage accumulation in fish.

### 4.3. Dietary MLE Was Associated with Intestinal Immune and Inflammatory Homeostasis

Systemic innate immune status and intestinal inflammatory homeostasis jointly determine fish intestinal health and stress resistance. Serum complement components (C3, C4) and immunoglobulin IgM are pivotal indicators reflecting the overall non-specific immune capacity of aquatic animals, while the balance of intestinal pro-inflammatory and anti-inflammatory cytokines sustains epithelial barrier stability and mucosal immune tolerance [56,57]. In Amur sturgeon, intestinal antioxidant defense and immune function are closely coupled with spiral valve intestinal mucosal integrity; impaired intestinal barrier usually leads to declined immune competence and aggravated oxidative injury [33]. Mulberry leaf bioactive components including flavonoids and polysaccharides have been widely reported to enhance innate immune responses and alleviate intestinal inflammatory injury in fish [46]. In the present study, moderate MLE supplementation significantly elevated serum C3, C4 and IgM concentrations in the MLE2 and MLE4 groups, indicating that appropriate MLE inclusion was associated with improved systemic immune-related parameters in juvenile *Acipenser schrenckii*. Furthermore, the physiological efficacy of mulberry leaf materials is strongly dependent on processing approaches; modified mulberry leaf products have been reported to exert distinct regulatory effects on fish immune function compared with raw mulberry leaf meal [41].

At the intestinal transcriptional level, the pro-inflammatory genes *tnfa* and *il1β*, as well as the immunoregulatory gene *tgfβ*, remained relatively stable across all treatments. The unchanged expression of pro-inflammatory markers suggested that graded MLE supplementation did not trigger abnormal intestinal inflammatory activation, consistent with its use as a functional feed ingredient. Notably, the core anti-inflammatory cytokine gene *il10* was significantly upregulated in the MLE2 and MLE4 groups, peaking in the MLE4 group. As a vital negative immune regulator, IL-10 restrains excessive inflammatory cascades and protects intestinal epithelial integrity [56]. The increased *il10* expression further suggested that moderate MLE supplementation was associated with a more balanced intestinal anti-inflammatory status and stabilized local immune homeostasis. Comparable improvements in intestinal health after mulberry leaf supplementation have also been observed in spotted sea bass [18].

Consistent with the quadratic antioxidant responses observed above, the immune-related effects of MLE displayed obvious dose-dependent attenuation at high dosage. The beneficial changes in serum immune indices and elevated *il10* expression diminished in the MLE6 group, suggesting that excessive MLE inclusion was associated with disrupted immune regulation and weakened intestinal protective effects. Collectively, moderate dietary MLE was associated with enhanced systemic innate immune-related parameters and intestinal anti-inflammatory homeostasis, whereas high-dose supplementation failed to sustain such physiological benefits. These findings further support a preliminary optimal MLE dosage range for sturgeon cultivation, which requires validation in future dose–response studies.

### 4.4. MLE Was Associated with Taxon-Specific Changes in Intestinal Microbiota and Host Metabolic Profiles

Intestinal microbial communities of aquatic species are co-regulated by host intrinsic factors and external environmental cues, among which dietary ingredients act as critical drivers shaping gut microbial composition and functionality [58]. Metabolomics represents a powerful tool to unravel diet-induced metabolic alterations in aquatic animals. Intestinal metabolomic profiles can reflect the efficiency of nutrient digestion and absorption, as well as bidirectional metabolic crosstalk between the host and gut microbiota [59,60]. Intestinal microbiota constitutes a vital regulatory hub linking diet, intestinal epithelial function, systemic metabolism and immune homeostasis in teleost fish [61]. Plant-derived bioactive substances can selectively modulate intestinal microbial community assembly and further shape intestinal metabolic profiles via microbe–host crosstalk [62].

In the present study, dietary MLE was associated with taxon-specific shifts in the intestinal microbiota of juvenile *Acipenser schrenckii*, although overall microbial community structure and alpha diversity were not significantly altered, as indicated by the non-significant Adonis (*p* = 0.228) and PERMDISP (*p* = 0.339) results. At the genus level, moderate MLE supplementation increased the relative abundances of beneficial taxa including *Priestia* and *Bacillus_P*. Members of these genera can produce extracellular enzymes, facilitate carbohydrate fermentation, and generate bioactive metabolites, which may contribute to improved intestinal digestive function and the suppression of potential pathogenic bacteria [63,64]. In addition, MLE was associated with the specific enrichment of the glycolipid-metabolizing genus *Cetobacterium_A* at 2% and 4% supplementation levels. As a core functional taxon in the intestinal tract of teleost fish, *Cetobacterium* participates in carbohydrate fermentation and lipid catabolism, stabilizes intestinal mucosal integrity and hepatic lipid metabolism, and enhances host antiviral immunity [65,66,67]. The specific enrichment of *Cetobacterium_A* in MLE2 and MLE4 was correlated with reduced serum TC, TG, and Glu concentrations, as well as with changes in antioxidant and immune-related parameters in these two groups.

Metabolomic analysis further revealed that MLE intervention was associated with changes in intestinal metabolic profiles. Differentially accumulated metabolites were mainly enriched in pathways related to carbohydrate metabolism, lipid metabolism, and amino acid metabolism. Such metabolic alterations may be partially attributed to changes in specific microbial taxa, as microbial fermentation can produce short-chain fatty acids, bile acid derivatives, and other signal molecules that regulate epithelial energy supply, antioxidant status, and inflammatory response [68,69]. Spearman correlation analysis uncovered significant correlations between characteristic microbial taxa and differential metabolites, suggesting a potential microbiota–metabolite association in the sturgeon intestine. It should be emphasized that these correlations are exploratory, were not corrected for multiple testing, and indicate statistical association rather than causal relationships. Further intervention and mechanistic studies are required to establish causality.

Combined with the foregoing physiological observations, these taxon-specific microbial alterations and associated metabolic changes may help explain the intestinal protective effects of MLE. The enriched beneficial microbes and shifted metabolic landscape may act together to reinforce intestinal barrier integrity, modulate Nrf2/Keap1-related antioxidant capacity, and maintain balanced intestinal inflammatory status. However, it should be emphasized that overall microbial community structure was not significantly altered, and the observed effects were mainly taxon-specific rather than a global remodeling of the microbiome. These findings suggest that the microbiota–metabolite–immunity axis may provide a plausible exploratory framework for the physiological changes observed in the MLE2 and MLE4 groups: the enrichment of *Cetobacterium_A* and associated metabolic changes were associated with Nrf2-related antioxidant responses and IL-10-mediated anti-inflammatory regulation. Nevertheless, consistent with the dose-dependent trend observed in antioxidant and immune indices, the effects of MLE on specific bacterial taxa and metabolic homeostasis were weakened at the highest inclusion level. This phenomenon further suggests that excessive MLE supplementation may disturb balanced microbe–host interactions, partially weakening the physiological benefits for intestinal health. Collectively, moderate MLE supplementation was associated with changes in the relative abundance of key intestinal bacterial taxa and in host metabolic profiles; this coordinated microbiota–metabolite network may represent one of several factors associated with the intestinal benefits observed in sturgeon.

Future studies should investigate the long-term effects of MLE supplementation across different life stages and under various stress conditions, as well as the potential synergistic effects of MLE combined with other functional feed additives.

## 5. Limitations

This study has several limitations. First, because all mulberry leaf-containing diets included the multi-enzyme premix and no diet contained mulberry leaf alone, the present design cannot fully separate the effects of mulberry leaf from those of the enzyme premix; a non-enzymatically treated mulberry leaf control is needed in future studies. Second, although *keap1* was downregulated and *cat* was upregulated in MLE2, transcript-level data cannot fully confirm activation of the Nrf2/Keap1 signaling cascade, and multiple-testing correction was not applied to the multi-omics correlation analyses; these results should therefore be interpreted as exploratory, and protein-level and FDR-corrected analyses are warranted. Third, the microbiota–metabolite correlations indicate association rather than causation. Finally, the present study did not include antibiotic comparison, bacterial challenge, disease-resistance testing, or antibiotic-residue assessment.

## 6. Conclusions

Moderate dietary supplementation (2–4%) with mulberry leaf powder plus a multi-enzyme premix (MLE) maintained normal growth and was associated with improved antioxidant capacity, immune-related parameters, and intestinal health in juvenile *Acipenser schrenckii*. These benefits may be linked to the coordinated regulation of the Nrf2/Keap1 antioxidant pathway, IL-10-mediated anti-inflammatory homeostasis, and taxon-specific changes in gut microbial composition—particularly the enrichment of beneficial glycolipid-metabolizing taxa such as *Cetobacterium_A*. However, the effects exhibited typical hormetic dose–response characteristics, with high-dose MLE (6%) attenuating or reversing these benefits. Based on quadratic regression analyses, a preliminary estimated optimal dietary inclusion range of 3.5–4.5% MLE is suggested; however, this estimate is based on only four dietary levels and should be validated in future dose–response studies before being considered a practical recommendation. These findings provide theoretical insights into the microbiota–metabolite–immunity axis in fish and a basis for further development of functional aquafeeds.

## Figures and Tables

**Table 1:** Effects of dietary MLE supplementation on growth performance and morphometric indices of juvenile Amur sturgeon (*Acipenser schrenckii*).

Items	Groups				SEM	*p*-Value		
	CK	MLE2	MLE4	MLE6		ANOVA	Linear	Quadratic
Growth performance								
FBW (g)	421.05 ^a^	414.25 ^ab^	393.33 ^ab^	385.38 ^b^	7.5017	0.017	0.0013	0.9395
WGR (%)	112.48 ^a^	107.83 ^a^	98.54 ^ab^	92.33 ^b^	3.4077	0.0054	0.0002	0.8179
SGR (%/d)	1.08 ^a^	1.04 ^a^	0.98 ^ab^	0.93 ^b^	0.0239	0.0044	0.0002	0.7926
FCR	1.26 ^b^	1.33 ^ab^	1.44 ^ab^	1.47 ^a^	0.0478	0.0278	0.0023	0.6566
FR (%)	1.28	1.32	1.34	1.33	0.0232	0.3624	0.1209	0.332
Morphometric parameters								
CF (g/cm^3^)	0.76	0.76	0.69	0.72	0.0119	0.0657	0.0739	0.3295
HSI (%)	3.77	3.55	3.35	3.26	0.1812	0.059	0.0095	0.5503
VSI (%)	10.22	10.26	9.95	10.18	0.3152	0.4589	0.4514	0.9002

FBW = final body weight; WGR = weight gain rate; SGR = specific growth rate; FCR = feed conversion rate; FR = feeding rate; CF = condition factor; HSI = hepatosomatic index; VSI = viscerosomatic index. ^a,b^ Within a row, means without a common superscript letter differ at *p* < 0.05, *n* = 4.

**Table 2:** Effects of dietary MLE supplementation on serum biochemical, antioxidant, and immune indices of juvenile Amur sturgeon (*Acipenser schrenckii*).

Items	Groups				SEM	*p*-Value		
	CK	MLE2	MLE4	MLE6		ANOVA	Linear	Quadratic
Energy substrates indicators								
TC (mmol/L)	1.98 ^a^	1.27 ^b^	1.21 ^b^	1.42 ^b^	0.066	0.0001	0.0352	<0.0001
TG (mmol/L)	6.66 ^a^	4.29 ^b^	4.21 ^b^	4.73 ^b^	0.131	<0.0001	0.0226	<0.0001
GLU (mmol/L)	4.01 ^a^	2.44 ^c^	3.39 ^ab^	2.92 ^bc^	0.160	0.0007	0.1783	0.1302
Antioxidant indicators								
T-AOC (mmol/L)	0.26 ^b^	0.31 ^a^	0.35 ^a^	0.32 ^a^	0.012	0.0006	0.0077	0.0017
T-SOD (U/mL)	107.78 ^c^	114.76 ^bc^	136.12 ^a^	132.01 ^ab^	4.446	0.0019	0.0009	0.2813
MDA (nmol/mL)	2.94 ^a^	2.70 ^a^	2.19 ^b^	2.36 ^b^	0.068	<0.0001	0.0002	0.0383
Immunologic indicators								
C3 (mg/dL)	11.67 ^c^	14.71 ^ab^	14.96 ^a^	14.34 ^b^	0.118	<0.0001	0.0008	<0.0001
C4 (mg/dL)	2.31 ^c^	2.69 ^b^	2.90 ^a^	2.89 ^ab^	0.048	<0.0001	<0.0001	0.0005
IgM (mg/dL)	9.47 ^b^	21.94 ^a^	22.08 ^a^	21.05 ^a^	0.342	<0.0001	0.0003	<0.0001

^a–c^ Within a row, means without a common superscript letter differ at *p* < 0.05, energy substrates indicators (*n* = 3), antioxidant indicators (*n* = 4), immunologic indicators (*n* = 5).

## Data Availability

The raw data supporting the conclusions of this article will be made available by the authors on request.

## Supplementary Materials

The following supporting information can be downloaded at: https://www.mdpi.com/article/10.3390/foods15183319/s1, Table S1: Enzyme components of exogenous enzyme preparation; Table S2: Formulation and compositions of experimental diets (%); Table S3: Primer sequences for real-time PCR; Table S4: Results of pairwise permutational multivariate dispersion (PERMDISP); Table S5: OPLS-DA model parameter table; Figure S1: Permutation-test outputs of OPLS-DA models under positive (POS) and negative (NEG) ionization modes.

## Abbreviations

The following abbreviations are used in this manuscript:

MLE	enzymatically treated mulberry leaf
TG	triglyceride
TC	total cholesterol
GLU	glucose
T-AOC	total antioxidative capability
T-SOD	total superoxide dismutase
MDA	malondialdehyde
C3	complement 3
C4	complement 4
IgM	immunoglobulin m
Vh	villus height
Cd	crypt depth
*keap1*	kelch-like ech-associated protein 1
*nrf2*	nuclear factor erythroid 2-related factor 2
*mafk*	maf bzip transcription factor k
*maf**g*	maf bzip transcription factor g
*cat*	catalase
*tnfα*	tumor necrosis factor α
*il1β*	interleukin 1β
*tgfβ*	transforming growth factor β1
*il10*	interleukin 10

## References

[1] Bemis W., Findeis E., Grande L. (2002). An overview of Acipenseriforms. Environ. Biol. Fishes.

[2] Chen R., Liu Z., Wang J., Jin W., Abdu H.I., Pei J., Wang Q., Abd El-Aty A.M. (2022). A review of the nutritional value and biological activities of sturgeon processed byproducts. Front. Nutr..

[3] Dudu A., Georgescu S.E. (2024). Exploring the Multifaceted Potential of Endangered Sturgeon: Caviar, Meat and By-Product Benefits. Animals.

[4] Chan J.C.F., Lam B.Y.K., Dudgeon D., Liew J.H. (2025). Global consequences of dam-induced river fragmentation on diadromous migrants: A systematic review and meta-analysis. Biol. Rev..

[5] White S.L., Fox D.A., Beridze T., Bolden S.K., Johnson R.L., Savoy T.F., Scheele F., Schreier A.D., Kazyak D.C. (2023). Decades of Global Sturgeon Conservation Efforts Are Threatened by an Expanding Captive Culture Industry. Fisheries.

[6] Onofri L., Pulcini D., Martini A., Martinoli M., Napolitano R., Tonachella N., Capoccioni F. (2024). Economic Analysis of Sturgeon Farming and Caviar Production: A Case Study of an Italian Fish Farm. Water.

[7] Degani G., Yom Din G. (2022). A Business Analysis of Innovations in Aquaculture: Evidence from Israeli Sturgeon Caviar Farm. Businesses.

[8] Bronzi P., Chebanov M., Michaels J.T., Wei Q., Rosenthal H., Gessner J. (2019). Sturgeon meat and caviar production: Global update 2017. J. Appl. Ichthyol..

[9] Ministry of Agriculture and Rural Affairs of the People’s Republic of China (2024). Reply to Proposal No. 7319 of the 2nd Session of the 14th National People’s Congress.

[10] Dossou S., Dawood M.A.O., Zaineldin A.I., Abouelsaad I.A., Mzengereza K., Shadrack R.S., Zhang Y., El-Sharnouby M., Ahmed H.A., El Basuini M.F. (2021). Dynamical Hybrid System for Optimizing and Controlling Efficacy of Plant-Based Protein in Aquafeeds. Complexity.

[11] McClelland J.W., Norris J.M., Dominey-Howes D., Govendir M. (2022). Knowledge and perceptions of Australian postgraduate veterinary students prior to formal education of antimicrobial use and antimicrobial resistance. One Health.

[12] National Health Commission of the People’s Republic of China (2002). List of Items Used as Both Food and Medicine.

[13] Ministry of Agriculture and Rural Affairs of the People’s Republic of China (2012). Catalog of Feed Raw Materials.

[14] Liu Y.Y., Li Y.H., Xiao Y., Peng Y.L., He J.H., Chen C., Xiao D.F., Yin Y.L., Li F.N. (2021). Mulberry leaf powder regulates antioxidative capacity and lipid metabolism in finishing pigs. Anim. Nutr..

[15] So-In C., Sunthamala N. (2022). The effects of mulberry (*Morus alba* Linn.) leaf supplementation on growth performance, blood parameter, and antioxidant status of broiler chickens under high stocking density. Vet. World.

[16] Fu B., Zhou D., Qiu X., Zheng J., Yang Q., Xiao Y., Liao S., Li Q., Xing D. (2025). Mulberry leaf extract reduces damage by regulating the oxidative response, immune response and intestinal flora of largemouth bass (*Micropterus salmoides*) under chronic heat stress. Front. Mar. Sci..

[17] Wang Y., Jia J., Xu G., Ding L. (2025). Research Progress on Nutritional Characteristics of Mulberry Leaf and Its Application in Aquatic Feeds. Chin. J. Anim. Nutr..

[18] Zhou S.-S., Lin H., Kong L.-M., Ma J.-R., Long Z.-Y., Qin H.-H., Lin Y., Liu L.-H., Huang Z.-F., Li Z.-B. (2024). Mulberry leaf extract on antioxidative capacity and non-specific immune of *Lateolabrax maculatus*. Acta Hydrobiol. Sin..

[19] Mithul Aravind S., Wichienchot S., Tsao R., Ramakrishnan S., Chakkaravarthi S. (2021). Role of dietary polyphenols on gut microbiota, their metabolites and health benefits. Food Res. Int..

[20] van der Hee B., Wells J.M. (2021). Microbial Regulation of Host Physiology by Short-chain Fatty Acids. Trends Microbiol..

[21] Sankarganesh P., Bhunia A., Ganesh Kumar A., Babu A.S., Gopukumar S.T., Lokesh E. (2025). Short-chain fatty acids (SCFAs) in gut health: Implications for drug metabolism and therapeutics. Med. Microecol..

[22] Vargas R.A., Soto-Aguilera S., Parra M., Herrera S., Santibañez A., Kossack C., Saavedra C.P., Mora O., Pineda M., Gonzalez O. (2023). Analysis of microbiota-host communication mediated by butyrate in Atlantic salmon. Comput. Struct. Biotechnol. J..

[23] Ebrahimi R., Mohammadpour A., Medoro A., Davinelli S., Saso L., Miroliaei M. (2025). Exploring the links between polyphenols, Nrf2, and diabetes: A review. Biomed. Pharmacother..

[24] Lastra J.M., Andrés C.M., Munguira E.B., Juan C.A., Lebeña E.P. (2026). Dietary Polyphenols as Modulators of Redox Signalling: From the Antioxidant-Pro-Oxidant Continuum to Clinical Translation (2015–2025). Curr. Issues Mol. Biol..

[25] Ma W., Zeng W., Zhang D., Zhou Y., Huang Y., Hong Y. (2025). Oxidative Stress in Aquaculture: Pathogenic Mechanisms and Preventive Strategies in Farmed Aquatic Animals. Curr. Issues Mol. Biol..

[26] Long L., Zhang H., Ni Q., Liu H., Wu F., Wang X. (2019). Effects of stocking density on growth, stress, and immune responses of juvenile Chinese sturgeon (*Acipenser sinensis*) in a recirculating aquaculture system. Comp. Biochem. Physiol. Part C Toxicol. Pharmacol..

[27] Ning L., Gao L., Zhou W., Liu S., Chen X., Pan Q. (2021). Beneficial effects of dietary mulberry leaf along with multi-enzyme premix on the growth, immune response and disease resistance of golden pompano Trachinotus ovatus. Aquaculture.

[28] Saleh E.S.E., Tawfeek S.S., Abdel-Fadeel A.A.A., Abdel-Daim A.S.A., Abdel-Razik A.-R.H., Youssef I.M.I. (2022). Effect of dietary protease supplementation on growth performance, water quality, blood parameters and intestinal morphology of Nile tilapia (*Oreochromis niloticus*). J. Anim. Physiol. Anim. Nutr..

[29] Xu S.-D., Zheng X., Dong X.-J., Ai Q.-H., Mai K.-S. (2022). Beneficial effects of phytase and/or protease on growth performance, digestive ability, immune response and muscle amino acid profile in low phosphorus and/or low fish meal gibel carp (*Carassius auratus gibelio*) diets. Aquaculture.

[30] Picone G., Balling Engelsen S., Savorani F., Testi S., Badiani A., Capozzi F. (2011). Metabolomics as a Powerful Tool for Molecular Quality Assessment of the Fish *Sparus aurata*. Nutrients.

[31] Bi B., Yuan Y., Zhao Y., He M., Song H., Kong L., Gao Y. (2023). Effect of crowding stress on growth performance, the antioxidant system and humoral immunity in hybrid sturgeon. Aquac. Rep..

[32] Yang S., Zhang C., Xu W., Li D., Feng Y., Wu J., Luo W., Du X., Du Z., Huang X. (2022). Heat Stress Decreases Intestinal Physiological Function and Facilitates the Proliferation of Harmful Intestinal Microbiota in Sturgeons. Front. Microbiol..

[33] Ng W.K., Hung S.S.O. (1995). Estimating the ideal dietary indispensable amino acid pattern for growth of white sturgeon, *Acipenser transmontanus* (Richardson). Aquac. Nutr..

[34] Wei H.C., Chen P., Liang X.F., Yu H.H., Wu X.F., Han J., Luo L., Gu X., Xue M. (2019). Plant protein diet suppressed immune function by inhibiting spiral valve intestinal mucosal barrier integrity, anti-oxidation, apoptosis, autophagy and proliferation responses in amur sturgeon (*Acipenser schrenckii*). Fish Shellfish Immunol..

[35] Bolyen E., Rideout J.R., Dillon M.R., Bokulich N.A., Abnet C.C., Al-Ghalith G.A., Alexander H., Alm E.J., Arumugam M., Asnicar F. (2019). Reproducible, interactive, scalable and extensible microbiome data science using QIIME 2. Nat. Biotechnol..

[36] Rognes T., Flouri T., Nichols B., Quince C., Mahé F. (2016). VSEARCH: A versatile open source tool for metagenomics. PeerJ.

[37] Mengucci C., Nissen L., Picone G., Malpuech-Brugère C., Orfila C., Ricciardiello L., Bordoni A., Capozzi F., Gianotti A. (2022). K-Clique Multiomics Framework: A Novel Protocol to Decipher the Role of Gut Microbiota Communities in Nutritional Intervention Trials. Metabolites.

[38] Wei H.C., Xing S.J., Chen P., Wu X.F., Gu X., Luo L., Liang X.F., Xue M. (2020). Plant protein diet-induced hypoimmunity by affecting the spiral valve intestinal microbiota and bile acid enterohepatic circulation in Amur sturgeon (*Acipenser schrenckii*). Fish Shellfish Immunol..

[39] Han Z., Wen M., Zhang H., Zhang L., Wan X., Ho C.T. (2022). LC-MS based metabolomics and sensory evaluation reveal the critical compounds of different grades of Huangshan Maofeng green tea. Food Chem..

[40] Mondal K., Kaviraj A., Mukhopadhyay P.K. (2012). Effects of partial replacement of fishmeal in the diet by mulberry leaf meal on growth performance and digestive enzyme activities of Indian minor carp *Labeo bata*. Int. J. Aquat. Sci..

[41] Wei Y., Huang J., Sun H., Feng Z., He Y., Chen Y., Lin S. (2023). Impact of different processing mulberry leaf on growth, metabolism and liver immune function of largemouth bass (*Micropterus salmoides*). Aquac. Rep..

[42] Kaviraj A., Mondal K., Mukhopadhyay P.K., Turchini G.M. (2013). Impact of Fermented Mulberry Leaf and Fish Offal in Diet Formulation of Indian Major Carp (*Labeo rohita*). Proc. Zool. Soc..

[43] Ma J., Yan X., Xu H., Pan L., Zhai X., Xue Y., Chen Y., Liu H., Zhao M., Luo L. (2024). Effects of mulberry leaf extract on growth, digestion, liver lipid metabolism and hypoglycaemic ability in mandarin fish (*Siniperca chuatsi*). Aquac. Rep..

[44] Feng Q.-F., Li Z.-F., Huang X.-Z., Li H., Zhai X.-L., Xue Y., Lin S.-M., Chen Y.-J., Luo L. (2021). Effects of dietary mulberry leaf extract and 1-deoxynojirimycin on growth, digestion and immunity capacity, and intestinal microorganism of Chinese giant salamander (*Andrias davidianus*). Acta Hydrobiol. Sin..

[45] Yao W., Wu X., Gao Y., Wu M., Lu S., Li X., Dong Y., Jin Z., Zhou Z. (2018). Effects of replacing fishmeal protein by hemoglobin powder protein on growth performance, food intake, feeding-related gene expression and gut histology of hybrid grouper (*Epinephelus fuscoguttatus* × *Epinephelus lanceolatus*) juveniles. Aquaculture.

[46] Zhou S., Huang Z., Lin H., Kong L., Ma J., Long Z., Qin H., Liu L., Lin Y., Li Z. (2023). Effects of mulberry leaf extract on the intestinal health of spotted sea bass (*Lateolabrax maculatus*). Front. Mar. Sci..

[47] Li Z., Chen X., Chen Y., Li W., Feng Q., Zhang H., Huang X., Luo L. (2020). Effects of dietary mulberry leaf extract on the growth, gastrointestinal, hepatic functions of Chinese giant salamander (*Andrias davidianus*). Aquac. Res..

[48] Wen L., Shi D., Zhou T., Tu J., He M., Jiang Y., Yang B. (2020). Identification of two novel prenylated flavonoids in mulberry leaf and their bioactivities. Food Chem..

[49] Yu Y., Chen Y., Shi X., Ye C., Wang J., Huang J., Zhang B., Deng Z. (2022). Hepatoprotective effects of different mulberry leaf extracts against acute liver injury in rats by alleviating oxidative stress and inflammatory response. Food Funct..

[50] Duan G.L., Wang C.N., Liu Y.J., Yu Q., Tang X.L., Ni X., Zhu X.Y. (2016). Resveratrol alleviates endotoxemia-associated adrenal insufficiency by suppressing oxidative/nitrative stress. Endocr. J..

[51] Almukainzi M., El-Masry T.A., Selim H., Saleh A., El-Sheekh M., Makhlof M.E.M., El-Bouseary M.M. (2023). New Insight on the Cytoprotective/Antioxidant Pathway Keap1/Nrf2/HO-1 Modulation by Ulva intestinalis Extract and Its Selenium Nanoparticles in Rats with Carrageenan-Induced Paw Edema. Mar. Drugs.

[52] Wu S., Liao X., Zhu Z., Huang R., Chen M., Huang A., Zhang J., Wu Q., Wang J., Ding Y. (2022). Antioxidant and anti-inflammation effects of dietary phytochemicals: The Nrf2/NF-κB signalling pathway and upstream factors of Nrf2. Phytochemistry.

[53] Habotta O.A., Dawood M.A.O., Kari Z.A., Tapingkae W., Van Doan H. (2022). Antioxidative and immunostimulant potential of fruit derived biomolecules in aquaculture. Fish Shellfish Immunol..

[54] Jantan I., Haque M.A., Arshad L., Harikrishnan H., Septama A.W., Mohamed-Hussein Z.-A. (2021). Dietary polyphenols suppress chronic inflammation by modulation of multiple inflammation-associated cell signaling pathways. J. Nutr. Biochem..

[55] Jiang S., Luo T., Huang Y., Yang Z., Yang Z., Meng X. (2026). Mulberry leaf polyphenols alleviate food allergy and moderate intestinal flora and lipid metabolism in allergic mice. J. Future Foods.

[56] Stosik M., Tokarz-Deptuła B., Deptuła W. (2023). Immunity of the intestinal mucosa in teleost fish. Fish Shellfish Immunol..

[57] Gomez D., Sunyer J.O., Salinas I. (2013). The mucosal immune system of fish: The evolution of tolerating commensals while fighting pathogens. Fish Shellfish Immunol..

[58] Zhou Z., Ringø E., Olsen R.E., Song S.K. (2018). Dietary effects of soybean products on gut microbiota and immunity of aquatic animals: A review. Aquac. Nutr..

[59] Nie Z., Xu X., Shao N., He J., Li P., Xu P., Hu J., Qin W., Wang B., Xu G. (2023). Integrative analysis of microbiome and metabolome reveals the linkage between gut microbiota and carp growth. Environ. Res..

[60] Ding X., Jin F., Xu J., Zhang S., Chen D., Hu B., Hong Y. (2022). The impact of aquaculture system on the microbiome and gut metabolome of juvenile Chinese softshell turtle (*Pelodiscus sinensis*). iMeta.

[61] Butt R.L., Volkoff H. (2019). Gut Microbiota and Energy Homeostasis in Fish. Front. Endocrinol..

[62] Chen X., Cai B., Wang J., Sheng Z., Yang H., Wang D., Chen J., Ning Q. (2021). Mulberry leaf-derived polysaccharide modulates the immune response and gut microbiota composition in immunosuppressed mice. J. Funct. Foods.

[63] Gao C., Yang W., Duan Y., Niu W., Wang Y., Qin M., Li Y. (2025). Dietary supplementation of Priestia sp. PPB30 attenuates intestinal colonization and inflammatory response induced by Aeromonas veronii in loach through interfering with the LuxS/AI-2 quorum sensing system. Fish Shellfish Immunol..

[64] Chen X., Liu S., Teame T., Luo J., Liu Y., Zhou Q., Ding Q., Yao Y., Yang Y., Ran C. (2025). Effect of Bacillus velezensis T23 solid-state fermentation product on growth, gut and liver health, and gut microbiota of common carp (*Cyprinus carpio*). Aquaculture.

[65] Meng D., Zhang Z., Teame T., Niemann B.E., Xia R., Xu S., Zhao Y., Yang Y., Ran C., Guan L.L. (2026). Host-driven hepatic conversion of gut microbiota-derived putrescine to spermidine mediates mannose’s protective effects against hepatic steatosis in zebrafish. iMeta.

[66] Wang A., Zhang Z., Ding Q., Yang Y., Bindelle J., Ran C., Zhou Z. (2021). Intestinal Cetobacterium and acetate modify glucose homeostasis via parasympathetic activation in zebrafish. Gut Microbes.

[67] Xie M., Xie Y., Li Y., Zhou W., Zhang Z., Yang Y., Olsen R.E., Ringø E., Ran C., Zhou Z. (2022). Stabilized fermentation product of *Cetobacterium somerae* improves gut and liver health and antiviral immunity of zebrafish. Fish Shellfish Immunol..

[68] Li T., Chiang J.Y.L. (2024). Bile Acid Signaling in Metabolic and Inflammatory Diseases and Drug Development. Pharmacol. Rev..

[69] Fu Y., Wang Y., Gao H., Li D., Jiang R., Ge L., Tong C., Xu K. (2021). Associations among Dietary Omega-3 Polyunsaturated Fatty Acids, the Gut Microbiota, and Intestinal Immunity. Mediat. Inflamm..

