# Supplementary material for: Mulberry Leaf Powder Supplemented with a Multi-Enzyme Premix Affects the Intestinal Microbiota–Metabolite–Immunity Axis and Nrf2/Keap1 Signaling in Juvenile Acipenser schrenckii: A Dose-Dependent Evaluation
Source: Foods. 2026 Sep 19;15(18):3319. doi: 10.3390/foods15183319 (PMC13606908; doi:10.3390/foods15183319)
Supplement: Supplementary file 1 [file foods-15-03319-s001.zip › foods-4524558-supplementary.pdf]

Supplementary Files

Table S1. Enzyme components of exogenous enzyme preparation.

Xylanase	Cellulase	Glucanase	Mannanase	Acid protease	Neutral protease	Alkaline protease	Medium-temperature α-amylase
3×10^4 U	2×10^4 U	1×10^5 U	2×10^3 U	3×10^3 U	1×10^4 U	6×10^3 U	3×10^5 mg/h

The enzyme activity values were determined in accordance with the enterprise standard of the manufacturer. U represents the enterprise-defined enzyme activity unit of VTR Bio-Tech.

Table S2. Formulation and compositions of experimental diets (%).

Ingredients	Experimental diets			
	**CK**	**MLE2**	**MLE4**	**MLE6**
Fish meal[1]	55.00	54.45	53.35	52.25
MLE	-	2.00	4.00	6.00
Wheat middling	18.00	15.67	10.35	5.00
Wheat gluten meal	19.00	19.00	22.00	25.00
Fish oil[1]	3.50	3.50	4.00	4.30
Soybean oil[1]	3.00	3.00	3.00	3.00
L-Lysine[2]	0.05	0.50	1.00	1.50
Yeast extract	1.00	1.00	1.00	1.00
DL-Met[2]	0.00	0.20	0.40	0.80
$Ca(H_2PO_4)_2$	0.05	0.08	0.10	0.15
Thr[2]	0.00	0.20	0.40	0.60
Mineral premix[3]	0.20	0.20	0.20	0.20
Vitamin premix[4]	0.20	0.20	0.20	0.20
Total	100	100	100	100
Analyzed nutrient composition (%, in dry matter basis)				
Dry matter	95.3	94.9	95.2	96.3
Organic matter	88.3	88.2	87.3	87.2
Crude protein	46.5	46.4	46.4	46.2
Crude lipid	11.4	11.2	11.6	11.9
Gross energy (MJ/kg)	19.7	19.5	19.4	20.0

[1] Fish meal was supplied by Pesquera Centinela Co., Lima, Peru. Fish oil and Soybean oil were supplied by Han Ye Biotechnology Co.,Ltd. Shangdong, China.

[2] Referencing the ideal AA model of white sturgeo, calculated the supplementation level of CAA.

[3] Mineral premixes (mg/kg diet): $CuSO_4\cdot5H_2O$ 10; $FeSO_4\cdot H_2O$ 300; ZnSO4·H_2O 200; $MnSO_4\cdot H_2O$ 80; KI (1%) 1.5; Na_2SeO_3 (10% Se) 10; $CoCl_2\cdot6H_2O$ (10% Co) 20; Zeolite 378.5.

[4] Vitamin premix (mg/kg diet): Vitamin A 20; Vitamin B1 12; Vitamin B2 10; Vitamin B6 15; Vitamin B12 (1%) 8; Niacinamide 100; Ascorbyl calcium phosphate (35%) 1000; Calcium pantothenate 40; Biotin (2%) 2; Folic acid 10; Vitamin E (50%) 400; Vitamin K3 20; Vitamin D3 10; Inositol 200; Choline chloride (50%) 4000; Corn protein powder 150:

Table S3. Primer sequences for real-time PCR.

Gene	Sequence 5′-3′	Target size (bp)	Tm (°C)	Efficiency values (%)
β-actin	F: CGGTTTCGCTGGAGATGATG<br>R: TCAGTGAGCAGGACGGGGTG	135	60	102
tnfα	F: AAAATGTCCAGCCATCAAAGCA<br>R: GTGAGGAATGATCCCGTTGGT	132	60	93
il1β	F: TGGTGGAGAAGAGAACGCAAGATG<br>R: GCGTCACTGCCTGTCTCATGG	114	60	105
tgfβ	F: TGCTCCTGTGCTCGGCTCTC<br>R: CAGCTCCATGTCCAGAGTCTTGC	80	60	109
il10	F: GCAAAGCTGAGGGAGCTGAG<br>R: CCCAGATTGACAGCTGGAGG	194	60	107
keap1	F: CCAGACCGGATAACGAGCTGAATC<br>R: GCCTGGCTTGTGAGACTCTGTATC	101	60	93
nrf2	F: TGGAAGTGTCCAGCCTGAGT<br>R: GTCGGCAGTCCCTTCTTTGG	138	60	101
mafg	F: ACAGGCGCCAGGTCTTAGTA<br>R: GAACTCAACCACTGCCACAA	155	58	95
mafk	F: AAATCAGCCGACCACAAGAC<br>R: ACTGGATGGACTGGCAAGAG	161	58	103
cat	F: TGGACACCGACACATGAATGGC<br>R: GGCGATGGCGTTGTACAGGTC	190	60	109

Tm, melting temperature; F, forward primer; R, reverse primer.
tnfα = Tumor Necrosis Factor α; *il1β* = Interleukin 1β; *tgfβ*= Transforming Growth Factor β; *il10* = Interleukin 10; *keap1* = Kelch-like ECH-associated protein 1; *nrf2* = Nuclear Factor Erythroid 2-Related Factor 2; *mafg* = MAF BZIP Transcription Factor G; *mafk* = MAF bZIP Transcription Factor K; *cat* = Catalase.

Table S4. Results of pairwise permutational multivariate dispersion (PERMDISP).

Pairwise comparison	p-value	q-value (FDR corrected)
CK_vs_MLE2	0.763	0.763
CK_vs_MLE4	0.469	0.616
CK_vs_MLE6	0.099	0.297
MLE2_vs_MLE4	0.457	0.616
MLE2_vs_MLE6	0.513	0.616
MLE4_vs_MLE6	0.084	0.297

q-value represents *P* value adjusted by FDR multiple testing correction. Significance threshold was set at $q < 0.05$. PERMDISP global test showed significant difference in community dispersion among FML groups ($P = 0.032$), while no pairwise comparison reached statistical significance after FDR correction.

Table S5. OPLS-DA model parameter table.

Groups	R^2Y(cum)	Q^2(cum)	Mode
CK VS MLE2	0.999	0.669	POS
CK VS MLE 4	0.992	0.809	POS
CK VS MLE 6	0.993	0.851	POS
CK VS MLE2	0.997	0.6	NEG
CK VS MLE 4	0.997	0.77	NEG
CK VS MLE 6	0.997	0.844	NEG

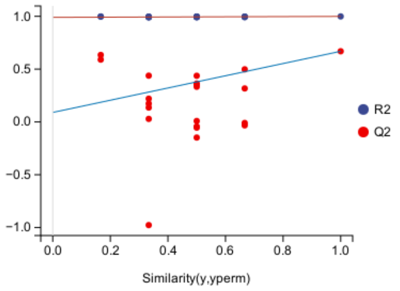

(**a**)

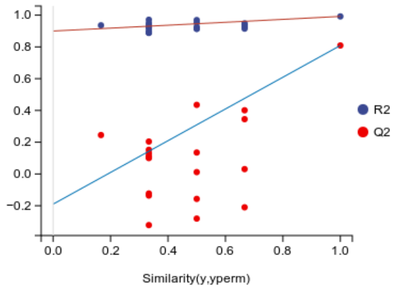

(**b**)

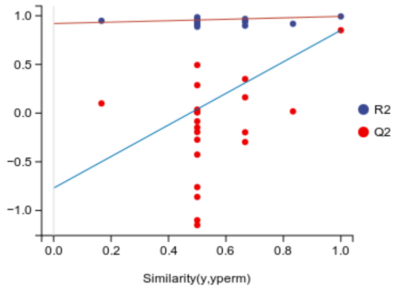

(c)

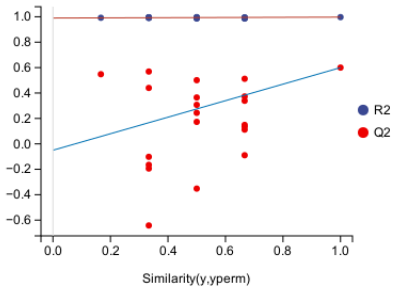

(**d**)

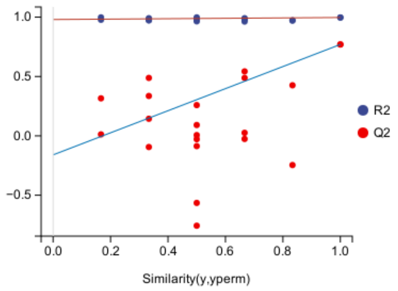

(**e**)

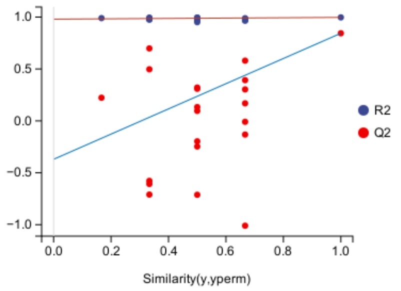

(**f**)

Figure. S1. Permutation-test outputs of OPLS-DA models under positive (POS) and negative (NEG) ionization modes. Panels (a–c) correspond to MLE2, MLE4 and MLE6 comparisons in POS mode; panels (d–f) represent MLE2, MLE4 and MLE6 comparisons in NEG mode. Permutation testing was applied to evaluate model significance and exclude over-fitting, confirming the robustness of each OPLS-DA discriminant model.
